# Evaluation of Dose Distribution in Intensity Modulated Radiosurgery for Lung Cancer under Condition of Respiratory Motion

**DOI:** 10.1371/journal.pone.0163112

**Published:** 2016-09-20

**Authors:** Mee Sun Yoon, Jae-Uk Jeong, Taek-Keun Nam, Sung-Ja Ahn, Woong-Ki Chung, Ju-Young Song

**Affiliations:** Department of Radiation Oncology, Chonnam National University Medical School, Gwangju, Korea; Technische Universitat Munchen, GERMANY

## Abstract

The dose of a real tumor target volume and surrounding organs at risk (OARs) under the effect of respiratory motion was calculated for a lung tumor plan, based on the target volume covering the whole tumor motion range for intensity modulated radiosurgery (IMRS). Two types of IMRS plans based on simulated respiratory motion were designed using humanoid and dynamic phantoms. Delivery quality assurance (DQA) was performed using ArcCHECK and MapCHECK2 for several moving conditions of the tumor and the real dose inside the humanoid phantom was evaluated using the 3DVH program. This evaluated dose in the tumor target and OAR using the 3DVH program was higher than the calculated dose in the plan, and a greater difference was seen for the RapidArc treatment than for the standard intensity modulated radiation therapy (IMRT) with fixed gantry angle beams. The results of this study show that for IMRS plans based on target volume, including the whole tumor motion range, tighter constraints of the OAR should be considered in the optimization process. The method devised in this study can be applied effectively to analyze the dose distribution in the real volume of tumor target and OARs in IMRT plans targeting the whole tumor motion range.

## Introduction

Radiosurgery for lung cancer can deliver higher dose per treatment than conventional radiotherapy, thereby reducing the number of days required for treatment. The applications are increasing with the development of accurate treatment equipment such as a linear accelerator (LINAC) that has a high dose rate photon beam and micro multi-leaf collimator (MMLC) [1–3]. Especially, intensity modulated radiosurgery (IMRS) adopting the intensity modulated radiation therapy (IMRT) technique that minimizes the side effects of the organs at risk (OARs) surrounding the tumor target has been applied effectively to clinical lung cancer treatment [4–7].

The effect of respiratory motion should be considered in the treatment plan for lung tumors, as this can introduce significant dosimetric errors in the radiosurgery process. Typical methods to resolve this issue can be classified into two types. The first is to reduce the treated volume by reducing the irradiated region using respiratory gating and active breathing control methods, which irradiate only at a specified time during the breathing cycle. The second method is to consider the exact treated volume based on the whole target motion range, and design a treatment plan with the internal target volume (ITV) delineated based on the four-dimensional computed tomography (4DCT) images.

The calculated dose in the plan and the real irradiated dose of the tumor target volume and OAR in the treatment might be comparable when using the respiratory gating method as long as the patient maintains a regular and stable breathing pattern. In contrast to this, for ITV-based treatment, where the whole tumor moving range is the target volume, there are difficulties in accurately estimating the dose for the real volume of tumor target and OAR. This is because the effect of respiratory motion cannot be perfectly simulated in the dose calculation process. Evaluation of the IMRT dose distribution in the respiratory motion range is more complicated as the radiation intensity varies with time and location in a complex manner [8–10]. The dose variation in the motional effect can be offset with average out during the many fraction treatments in a standard radiotherapy process. However, detailed evaluation of the real dose distribution, considering the respiratory motion effect, is required in the case of radiosurgery, where a very large fractional dose is used.

In this study, a practical method was devised to estimate dose distribution in the real volume of tumor target and OAR during the treatment process of lung IMRS based on ITV, which covers the whole respiratory motion range. A humanoid phantom and a dynamic phantom, which simulate respiratory motion, were used to acquire 4DCT data in the different motion ranges. In each set of the 4DCT data, ITV and OAR within the humanoid phantom were delineated and IMRS plans were designed. The delivery quality assurance (DQA) process for verifying the dosimetric accuracy was performed under the same conditions with real target motion. The dose distributions in the real volume of tumor target and OAR were evaluated based on the measured dosimetric data in the DQA process, and the difference between the calculated dose in the plan and the evaluated dose was analyzed considering the moving condition.

## Materials and Methods

### 4DCT acquisition and ITV delineation

**Fig 1:**
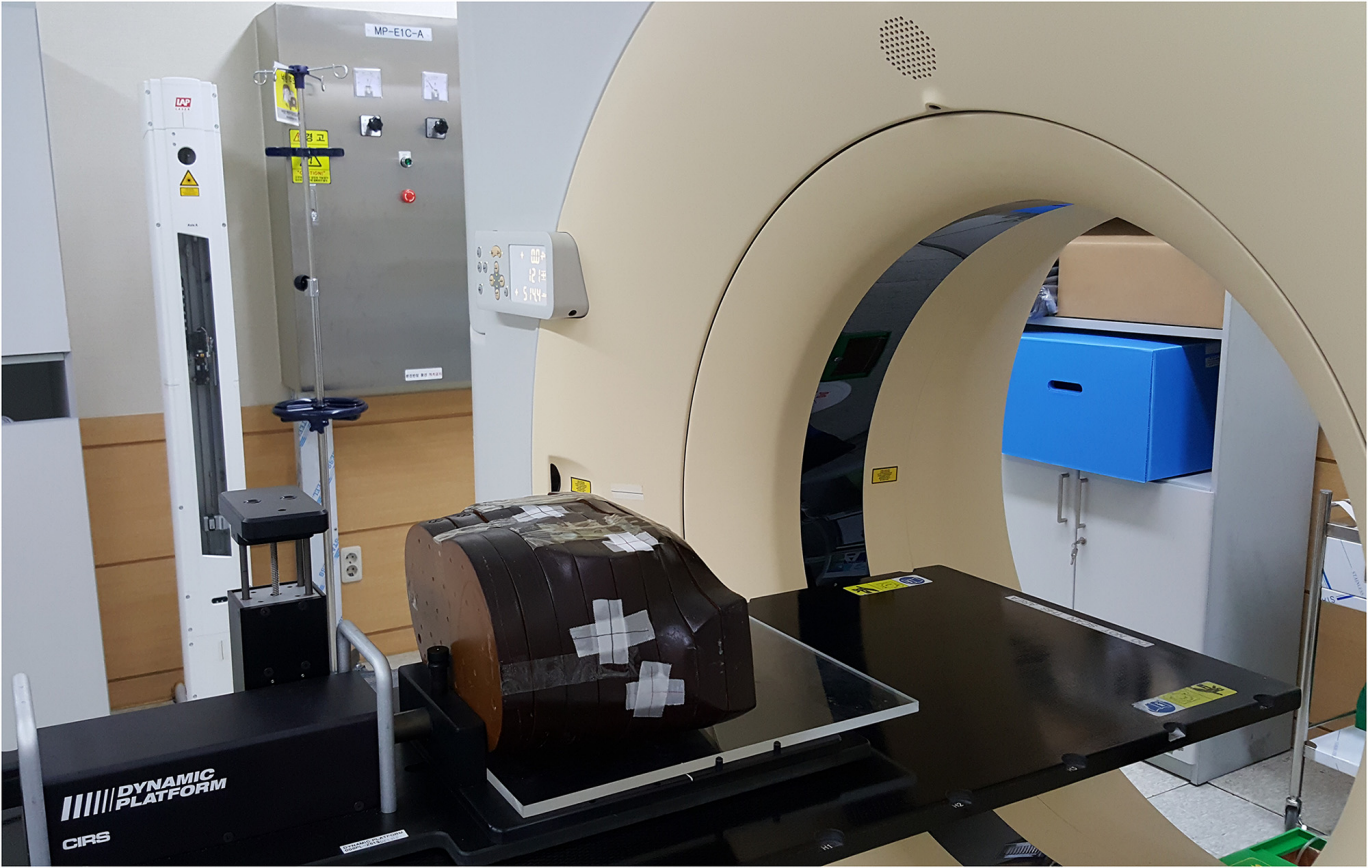
Humanoid chest phantom preparation on the moving phantom for the 4DCT acquisition of lung IMRS case.

The humanoid phantom for this study was prepared with recombined chest parts of the RANDO phantom (The Phantom Laboratory, Greenwich, NY, USA). The Dynamic Platform Model 008PL (CIRS, Norfolk, VA, USA), which can simulate respiratory motion, was used for the analysis of the respiratory motion effect. The 4DCT data of the humanoid chest phantom were acquired in four different motion ranges (1 cm, 2 cm, 3 cm, and 4 cm) and in a fixed motion cycle of 4 sec with a 2-mm slice thickness. The data were composed of 10 groups in total for each motion range, in accordance with the phase division of the respiratory cycle. The Brilliance CT Big Bore (Philips, Cleveland, OH, USA) was used for the acquisition of 4DCT data. Fig 1 shows the prepared phantom for 4DCT acquisition.

**Fig 2:**
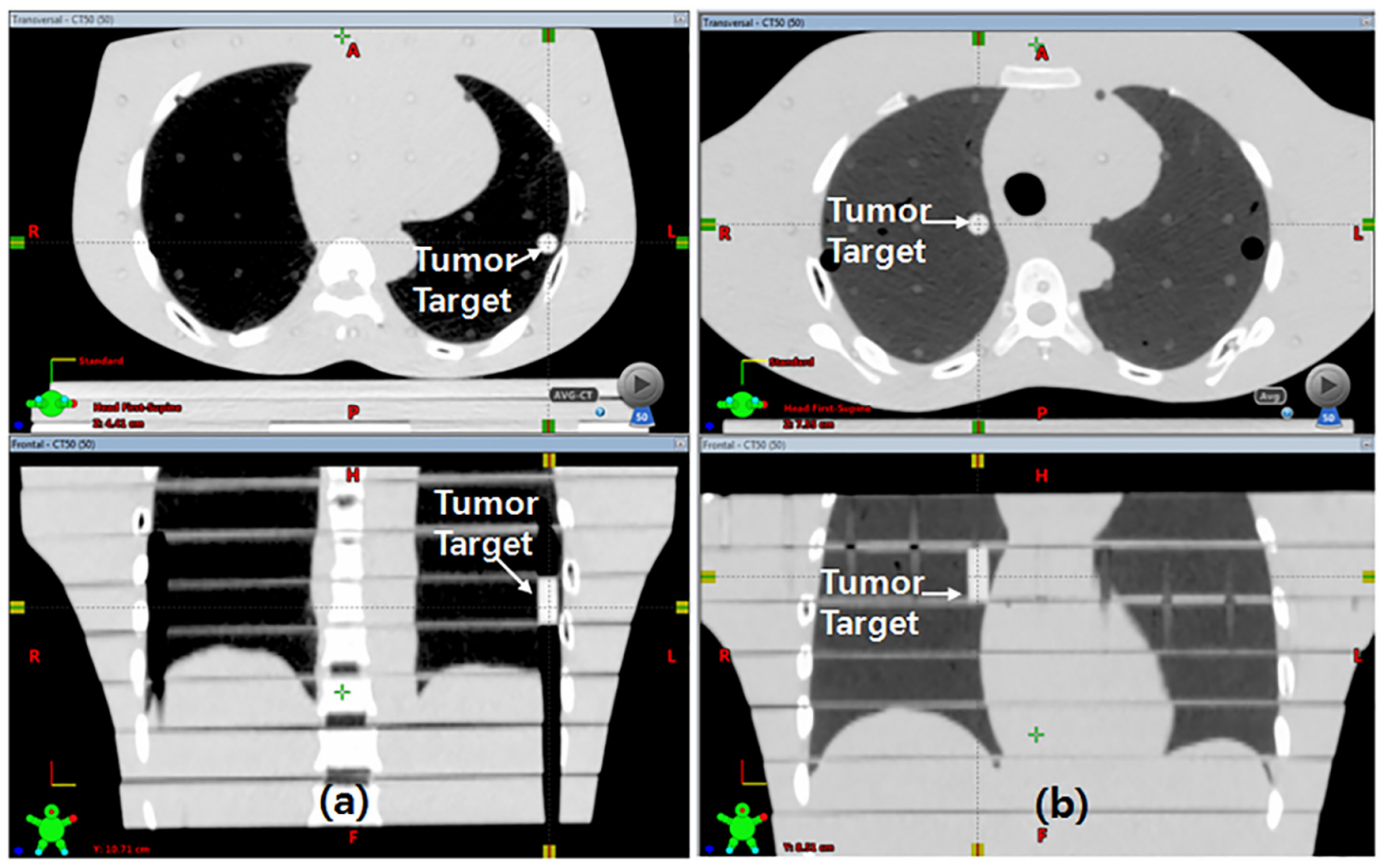
CT image of acrylic cylinder inserted for the simulation of lung tumor. (a) tumor target in the peripheral region of left lung, (b) tumor target in the central region.

An acrylic cylinder (1 cm in diameter and 2.5 cm in height) was inserted in the peripheral region of the left lung part and the central region of phantom separately to simulate a virtual lung tumor, which can be identified in the CT image as shown in Fig 2.

The tumor target and OAR were delineated manually at the 50% phase CT image and the structures were propagated automatically in the 9 other phase CT data sets using a deformable registration method. The ITV and planning organ at risk volume (PRV) were determined by combining the tumor target and OAR in each phase CT. The ITV was determined using an Eclipse (Varian, Palo Alto, CA, USA) planning system.

### Preparation of IMRS plan

The planning target volume (PTV) was created with a 2-mm margin to the ITV, and two types of IMRS plans were prepared for each of the four CT data sets, classified according to motion ranges. One was the RapidArc (Varian, Palo Alto, CA, USA) plan with two half rotation fields and the other was s conventional IMRT plan with fixed gantry angle beams (FB_IMRT). The fixed gantry angles used in this study were 0, 40, 60, 90, 130, 160, and 200° for the target (PTV_P_) in the peripheral left lung and 0, 200, 220, 245, 270, 310, and 340° for the target (PTV_C_) in the central region. The IMRS plans were created using an Eclipse planning system. The total prescription dose was 6,000 cCy for the PTV in 5 fractions and was optimized according to the constraints in Tables 1 and 2. Two different IMRS plans were created for each motion range CT data, and 16 IMRS plans were prepared.

**Table 1:** Dose constraints for the IMRS planning of peripheral lung tumor.

PTV_P_	V_5,880 cGy_ > 98%
Spinal Cord	D_max_ < 600 cGy
Aorta	D_max_ < 1,100 cGy
Chest Wall	D_max_ < 2,500 cGy
Heart	D_max_ < 1,800 cGy

**Table 2:** Dose constraints for the IMRS planning of central region lung tumor.

PTV_C_	V_5,880 cGy_ > 95%
Spinal Cord	D_max_ < 1,000 cGy
Aorta	D_max_ < 1,100 cGy
Bronchus	D_max_ < 2,800 cGy
Heart	D_max_ < 1,800 cGy

DQA plans for dosimetric verification were formulated for each IMRS plan. An ArcCHECK (SunNuclear, Melbourne, FL, USA) device was used for RapidArc DQA and a MapCHECK2 (SunNuclear, Melbourne, FL, USA) diode detector array was used for FB_IMRT DQA in each field. A clinical linear accelerator, Novalis Tx (Varian, Palo Alto, CA, USA), equipped with a 6MV flattening filter free (FFF) photon beam was used in this study.

### DQA dose measurement in the condition of respiratory motion

**Fig 3:**
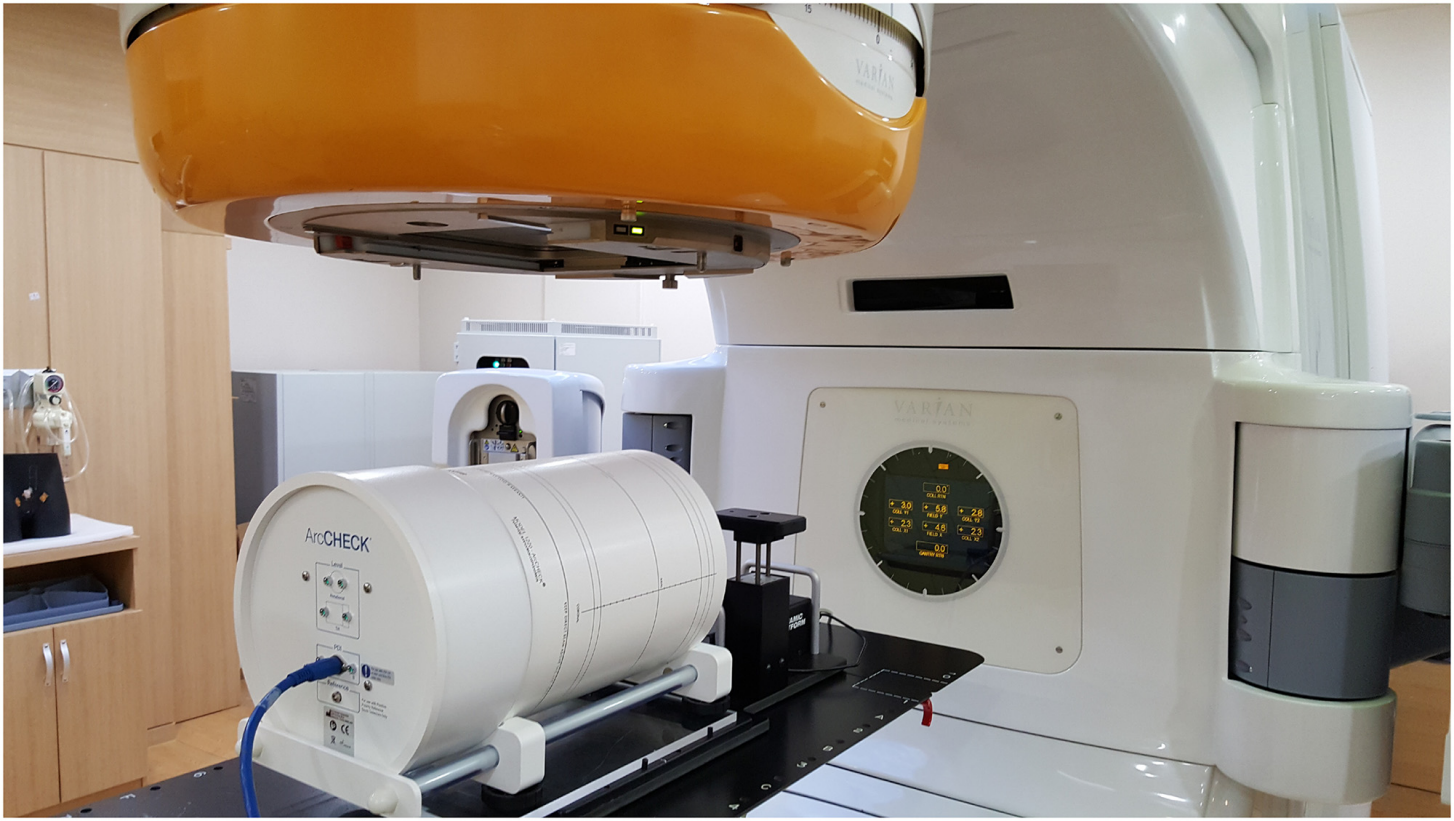
RapidArc DQA process using ArcCHECK under the respiratory motional environment.

**Fig 4:**
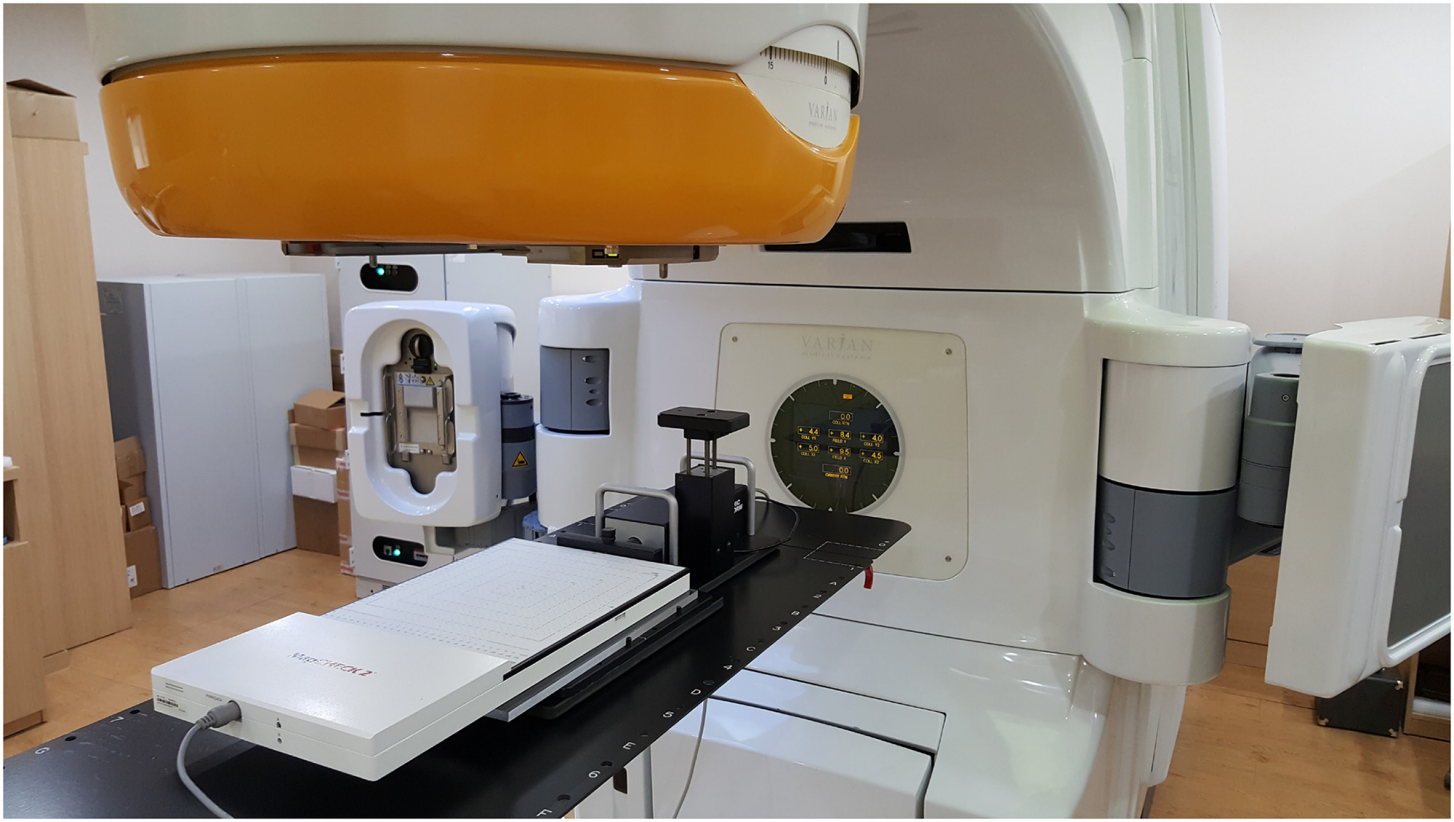
FB_IMRT DQA process using MapCHECK2 under the respiratory motional environment.

The IMRS DQA plans designed according to the motion ranges were executed using ArcCHECK and MapCHECK2 laid on the dynamic phantom, which simulates respiratory motion, as shown in Figs 3 and 4. The measurements in the 6-sec and 8-sec motion cycle were performed in addition to 4-sec cycle in the acquisition of 4DCT. Twelve sets of data were acquired with ArcCHECK measurements data and another twelve sets of data were acquired with MapCHECK2 measurements in the moving conditions. In this study, a single motion range in the superior-inferior (SI) direction was measured and was applied to the phantom simulation, as the dynamic phantom could move only in one direction, and the greatest changes in the respiratory motion usually occur in the SI direction.

All the measurements were made after aligning the treatment isocenter with the center of the DQA devices, (ArcCHECK and MapCHECK2). The absolute point dose in the central region of the tumor target was measured under moving conditions in order to analyze the dose variation compared with the calculated dose. Additional DQA plans were made with an ion chamber CC04 (IBA, Schwarzenbruck, Germany) inserted in the center of ArcCHECK and 48 absolute point doses were measured under moving conditions in the region of treatment isocenter.

### Dose recalculation in the real volume of tumor target and OARs

The conventional DQA process has is limited to a phantom material, and in order to overcome this limitation, special tools were developed to calculate the dose distributions in the bodies of patients using data from DQA processes [11–16]. The real dose distribution in the chest phantom was recalculated using the data from ArcCHECK and MapCHECK2 with the moving conditions. A 3DVH (SunNuclear, Melbourne, FL) program was used to recalculate the dose in the real volume of tumor target and OAR delineated inside the chest phantom.

**Fig 5:**
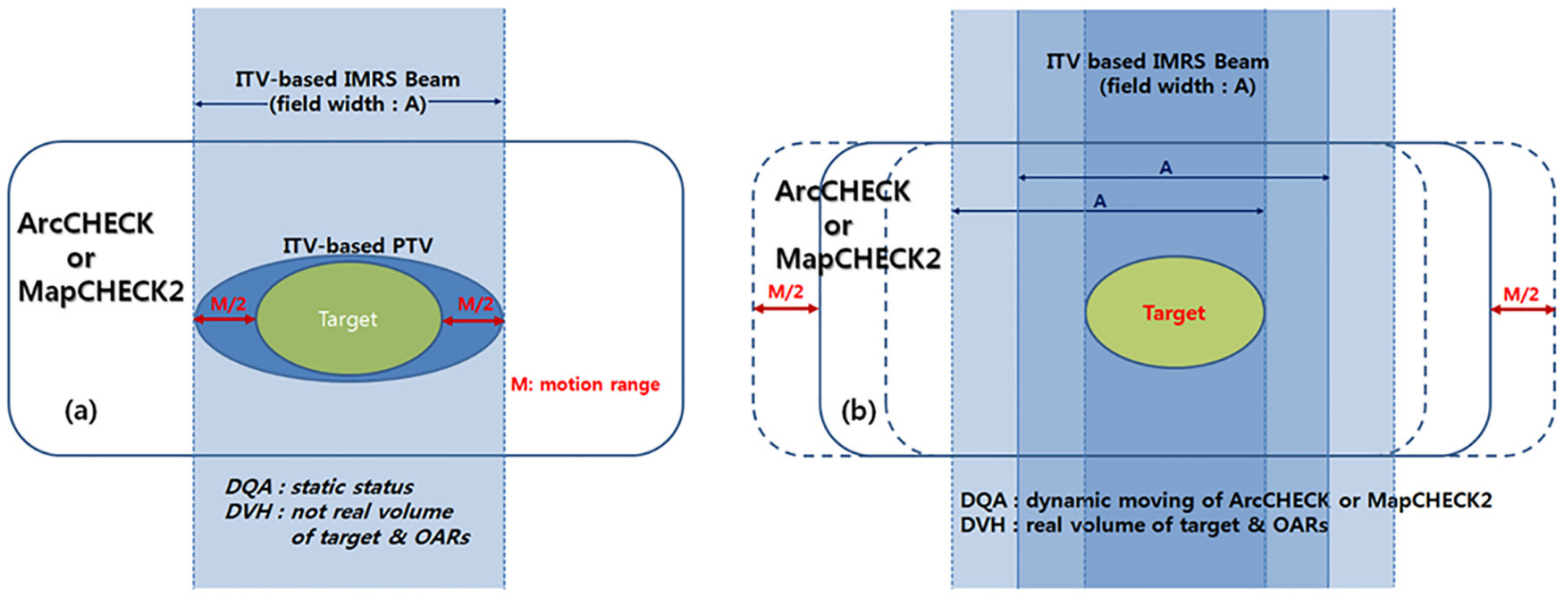
IMRS beam intensity which exhibit the same motion as the target and relatively equivalent condition of the target motion. (a) Real treatment condition in ITV-based IMRS, (b) relatively equivalent condition by the application measured dose data using moving ArcCHECK or MapCHECK2.

The variation of the dose distribution due to respiratory motion was deduced using the measured dosimetric data from the dynamic phantom. The IMRS beam intensity, which exhibited the same motion as the target, was realized with the measured data and a relatively equivalent condition of the motion of the target was assumed, as shown in Fig 5.

The mean dose and maximum dose were analyzed from the dose volume histogram (DVH) of the PTV and OAR in accordance with motion range and motion cycle, in addition to the lowest dose that irradiates all the PTV (D_100%_). All the volume of PTV and OAR applied in 3DVH were the real volume excluding the motion range which was considered in the ITV creation.

## Results

The PTV created in the CT data acquired with four different motion ranges are shown in Table 3. An increase in PTV with increase in motion range is seen.

**Table 3:** PTV volume created according to the motion range.

Motion Range	1cm	2cm	3cm	4cm
PTV_P_ volume [cm^3^]	6.1	10.4	11.1	13.9
PTV_C_ volume [cm^3^]	8.3	10.1	11.5	15.5

**Fig 6:**
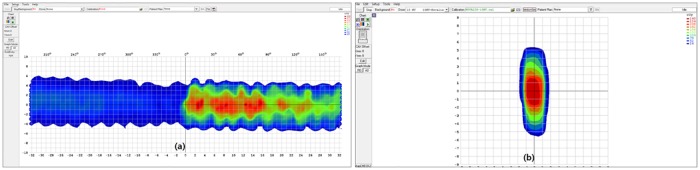
Example of DQA measurement under the moving condition. (a) ArcCHECK measurement, (b) MapCHECK2 measurement.

Fig 6 shows examples of IMRS DQA measurements with ArcCHECK for RapidArc, and MapCHECK2 for FB_IMRT with a motion range of 3 cm and cycle duration of 6 sec. The dose variation in the direction of SI, like a ripple shape in ArcCHECK measurement and the elongated dose shape in MapCHECK2 measurement due to the motion effect can be seen.

**Fig 7:**
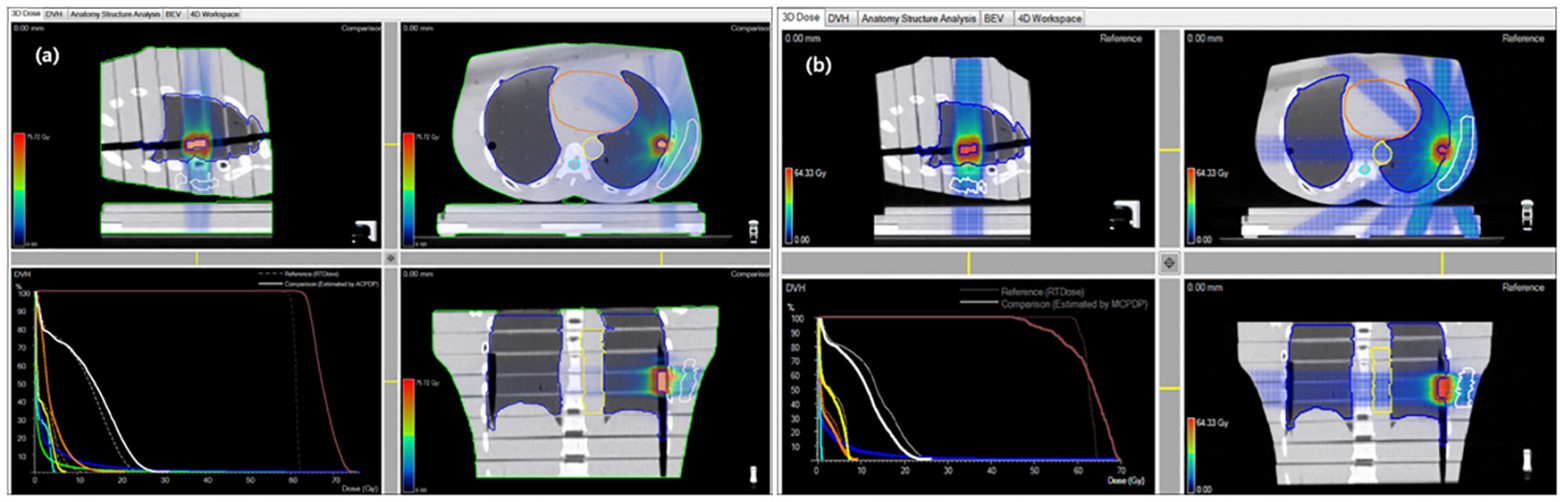
Example of 3DVH dose recalculation in chest phantom under the moving conditions. (a) RapidArc dose, (b) FB_IMRT dose.

The dose distribution in chest phantom is recalculated using 3DVH program with the data under the moving conditions based on the theory explained in Fig 5. An example of calculation is shown in Fig 7.

The typical dose values recalculated for the real volume of PTV and OAR according to the motion range and motion cycle are shown in Tables 4–7. These values are higher than the ones calculated in the IMRS plan.

**Table 4:** Recalculated RapidArc dose in the real volume of PTV_P_ and OARs under the moving condition.

Dose [Gy]	Motion Range	4cm			3cm			2cm			1cm		
	Motion Cycle	4sec	6sec	8sec	4sec	6sec	8sec	4sec	6sec	8sec	4sec	6sec	8sec
PTV_P_	D_100%_	60.7	59.8	60.2	54.5	57.5	55.1	56.1	57.1	56.4	57.8	57.5	57.6
	Mean	66.7	68.2	69.4	63.9	62.2	62.1	60.9	64.0	62.9	62.1	61.8	62.2
	Max	75.9	77.8	78.3	73.7	74.8	72.8	62.6	75.1	68.5	66.3	66.7	66.5
Cord	Mean	1.1	0.9	0.9	1.5	1.3	1.2	1.2	1.2	1.2	1.1	1.1	1.1
	Max	5.0	4.6	4.6	8.7	4.9	4.8	4.6	4.5	4.4	4.3	4.5	4.2
Chest	Mean	13.2	14.8	16.0	13.2	13.4	13.4	11.6	12.4	12.3	12.7	12.9	13.7
Wall	Max	34.8	30.8	33.7	38.3	33.4	33.6	27.1	31.2	32.0	36.9	38.6	46.2
Aorta	Mean	2.2	2.5	2.5	3.2	2.8	2.7	1.7	1.7	1.6	2.1	2.9	2.1
	Max	7.9	6.9	7.1	12.2	8.8	8.6	7.1	7.2	6.9	7.1	10.6	7.1
Lung	Mean	2.4	2.5	2.6	2.5	2.5	2.5	2.4	2.5	2.5	2.5	2.6	2.5
	Max	75.8	79.3	80.9	73.6	74.8	78.8	72.9	75.7	76.7	70.4	74.8	74.6
Heart	Mean	3.3	2.9	3.0	3.7	2.9	2.9	3.5	3.7	4.2	3.8	4.1	4.0
	Max	21.0	19.9	20.1	21.3	14.3	14.3	17.4	20.9	25.3	22.9	23.2	28.3

**Table 5:** Recalculated RapidArc dose in the real volume of PTV_C_ and OARs under the moving condition.

Dose [Gy]	Motion Range	4cm			3cm			2cm			1cm		
	Motion Cycle	4sec	6sec	8sec	4sec	6sec	8sec	4sec	6sec	8sec	4sec	6sec	8sec
PTV_C_	D_100%_	50.4	51.4	56.0	48.3	53.3	54.4	53.8	55.5	58.8	45.2	46.6	48.5
	Mean	78.9	77.4	80.0	72.6	76.1	77.7	68.9	70.5	74.7	65.1	67.1	69.5
	Max	85.5	87.1	91.4	81.9	86.9	88.7	82.2	83.6	88.5	79.9	82.5	88.1
Cord	Mean	2.8	2.8	3.3	2.7	3.2	3.2	4.1	4.0	4.1	3.3	3.4	3.5
	Max	10.9	10.9	16.5	10.6	16.1	16.3	20.3	20.9	21.6	11.4	11.6	12.3
Bron-	Mean	13.9	14.1	13.8	13.4	13.3	13.5	13.5	13.7	14.3	13.3	13.6	13.7
Chus	Max	47.7	48.5	51.1	45.9	48.9	49.8	47.6	49.8	52.3	47.4	48.7	50.4
Aorta	Mean	5.6	5.6	5.6	5.6	5.4	5.5	5.0	5.0	5.0	4.4	4.4	4.6
	Max	20.9	21.2	21.1	20.5	20.6	20.8	18.8	15.4	15.8	21.6	21.9	18.9
Lung	Mean	3.6	3.7	3.4	3.5	3.6	3.7	3.6	3.6	3.7	3.4	3.4	3.6
	Max	87.1	88.7	93.7	82.7	89.4	91.2	82.0	84.7	89.7	82.1	84.7	86.4
Heart	Mean	0.5	0.5	0.5	0.5	0.5	0.5	0.6	0.6	0.6	0.4	0.4	0.5
	Max	9.1	9.2	10.9	8.9	10.5	10.7	10.5	9.3	9.7	12.1	12.4	12.9

**Table 6:** Recalculated FB_IMRT dose in the real volume of PTV_P_ and OARs under the moving condition.

Dose [Gy]	Motion Range	4cm			3cm			2cm			1cm		
	Motion Cycle	4sec	6sec	8sec	4sec	6sec	8sec	4sec	6sec	8sec	4sec	6sec	8sec
PTV_P_	D_100%_	54.7	55.2	55.3	45.1	44.7	44.2	32.7	32.9	33.8	49.1	50.2	49.5
	Mean	62.5	62.7	62.6	62	62.1	61.1	57.5	58.2	57.7	61.8	61.9	62.0
	Max	65.1	65.5	65.4	69.5	69.6	68.2	67.7	67.8	67.3	65.2	65.2	65.6
Cord	Mean	0.5	0.5	0.5	0.3	0.3	0.3	0.3	0.4	0.4	0.4	0.4	0.3
	Max	1.3	1.2	1.2	0.9	0.8	0.8	0.9	2.0	1.9	1.0	1.0	1.1
Chest	Mean	15.9	16.0	16.0	10.5	10.5	10.5	10.0	10.1	10.0	12.6	12.7	12.6
Wall	Max	29.7	29.7	29.9	26.6	25.9	26.3	26.6	26.9	26.3	26.7	26.0	26.6
Aorta	Mean	3.9	4.0	3.9	2.9	2.9	2.8	2.3	2.3	2.3	2.9	2.9	2.9
	Max	8.7	8.7	8.9	7.2	7.3	6.9	9.1	9.4	9.2	7.2	7.2	7.4
Lung	Mean	3.1	3.1	3.1	2.2	2.2	2.2	2.2	2.2	2.2	2.7	2.7	2.7
	Max	63.9	64.2	64.2	68.1	68.9	66.6	66.9	66.7	66.3	64.4	64.3	64.6
Heart	Mean	2.2	2.2	2.2	1.6	1.6	1.6	1.9	1.9	1.9	2.1	2.2	2.1
	Max	13.4	13.3	13.7	9.1	9.0	8.3	8.8	8.8	8.7	9.5	9.3	9.4

**Table 7:** Recalculated FB_IMRT dose in the real volume of PTV_C_ and OARs under the moving condition.

Dose [Gy]	Motion Range	4cm			3cm			2cm			1cm		
	Motion Cycle	4sec	6sec	8sec	4sec	6sec	8sec	4sec	6sec	8sec	4sec	6sec	8sec
PTV_P_	D_100%_	51.5	51.7	51.8	49.0	49.6	50.8	50.6	50.9	51.6	52.9	52.6	52.6
	Mean	60.9	61.3	61.6	60.9	60.8	60.9	61.4	61.5	61.6	62.4	62.0	62.1
	Max	64.5	65.6	66.1	65.3	65.0	65.2	66.5	66.1	65.4	66.1	65.5	65.8
Cord	Mean	2.2	2.1	2.3	1.9	1.9	1.8	2.1	2.1	2.1	2.2	3.7	2.2
	Max	9.3	9.2	10.4	11.0	10.5	10.4	9.9	9.9	9.5	9.9	10.0	10.2
Bron-	Mean	11.9	11.9	38.5	11.4	11.4	11.5	11.4	11.4	11.4	11.9	11.9	11.9
chus	Max	36.9	37.1	61.6	29.5	29.2	28.7	34.2	34.6	34.5	33.4	33.3	33.4
Aorta	Mean	3.7	3.8	3.8	3.6	3.6	3.7	3.6	3.6	3.6	3.7	3.7	3.7
	Max	12.3	12.4	12.5	12.3	11.9	13.1	13.9	14.6	14.4	12.7	12.2	12.2
Lung	Mean	3.3	3.2	3.3	3.1	3.1	3.1	3.1	3.1	3.1	3.3	3.3	3.3
	Max	63.8	65.1	66.1	64.5	64.2	63.9	65.6	64.9	64.6	65.3	64.7	64.7
Heart	Mean	0.9	0.9	0.9	0.9	0.9	0.9	0.8	0.9	0.8	0.8	0.8	0.8
	Max	8.4	8.4	9.0	8.2	8.5	8.1	8.5	8.5	8.4	9.9	10.2	10.2

**Fig 8:**
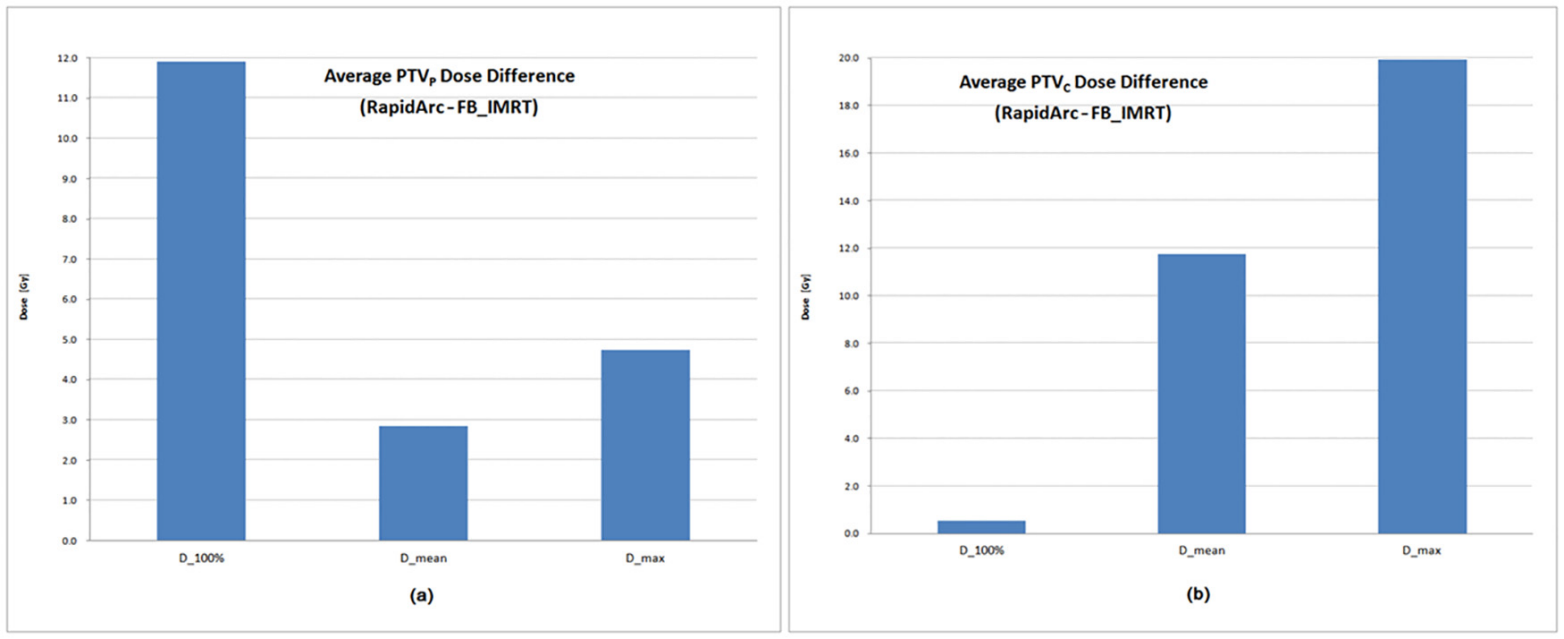
Average PTV dose difference between RapidArc and FB_IMRT. (a) tumor target in the peripheral region of left lung, (b) tumor target in the central region.

The increase of the dose in the PTV due to the motion effect was slightly different for RapidArc and FB_IMRT and Fig 8 shows the average difference in the recalculated PTV dose values between the two IMRS techniques. The dose in the RapidArc was higher than that in FB-IMRT with a difference of 7.4 Gy in the mean, 12.3 Gy in the maximum, and 6.3 Gy in D_100%_ on average.

**Fig 9:**
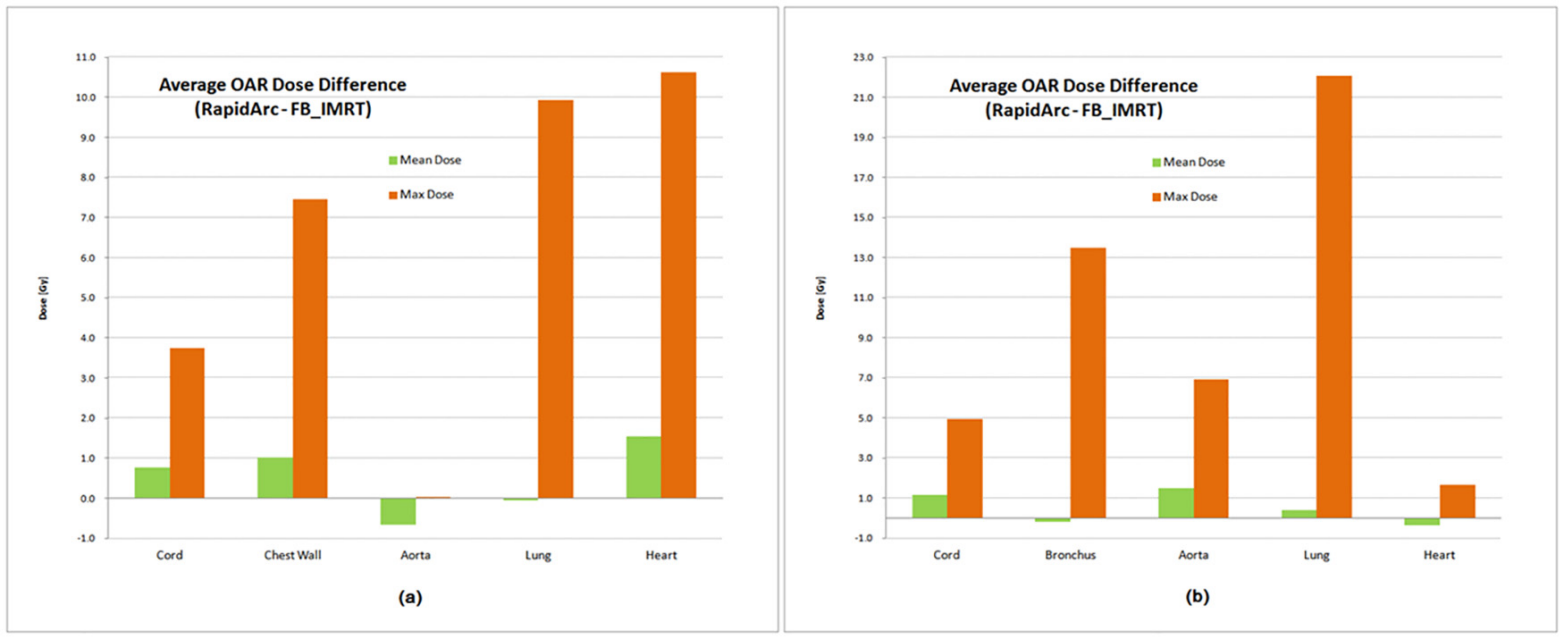
Average OAR dose difference between RapidArc and FB_IMRT. (a) tumor target in the peripheral region of left lung, (b) tumor target in the central region.

The recalculated dose distribution in OAR also showed different results compared to the calculated dose in the plan and the difference was dependant on the location and volume of OAR. In the dose comparison between the two IMRS techniques, the recalculated dose in the RapidArc was significantly higher (*p* < 10^−2^) than FB_IMRT except for the mean dose of lung and the dose of aorta in the IMRS for PTV_P_ and the mean dose of bronchus in the IMRS for PTV_C_ as shown in Fig 9.

The absolute isocenter point dose under moving conditions measured with an ion chamber inserted at the center of ArcCHECK is shown in Tables 8 and 9. The values are compared with dose calculation in a treatment planning system.

**Table 8:** Dose comparison between the RTP calculation and the real measurement at the isocenter of IMRS plans for tumor target in the peripheral region of left lung.

Motion Range	RapidArc Isocenter Point Dose [cGy]				FB_IMRT Isocenter Point Dose [cGy]			
	RTP	Measurement			RTP	Measurement		
		4sec	6sec	8sec		4sec	6sec	8sec
4cm	778.0	815.9	789.9	808.1	820.0	838.5	815.6	831.1
3cm	776.0	789.4	779.6	804.6	821.0	821.5	815.2	849.1
2cm	777.0	777.2	779.5	805.1	817.0	817.8	815.3	833.3
1cm	765.0	773.4	774.0	806.5	798.0	799.8	790.5	798.8

**Table 9:** Dose comparison between the RTP calculation and the real measurement at the isocenter of IMRS plans for tumor target in the central region.

Motion Range	RapidArc Isocenter Point Dose [cGy]				FB_IMRT Isocenter Point Dose [cGy]			
	RTP	Measurement			RTP	Measurement		
		4sec	6sec	8sec		4sec	6sec	8sec
4cm	915.4	959.4	962.2	955.5	918.0	958.4	951.7	954.6
3cm	908.0	952.5	945.9	944.9	938.2	947.7	960.1	945.9
2cm	910.8	965.8	936.4	934.5	926.4	947.8	942.1	946.8
1cm	899.4	945.9	943.0	930.7	930.5	950.6	951.6	953.4

**Fig 10:**
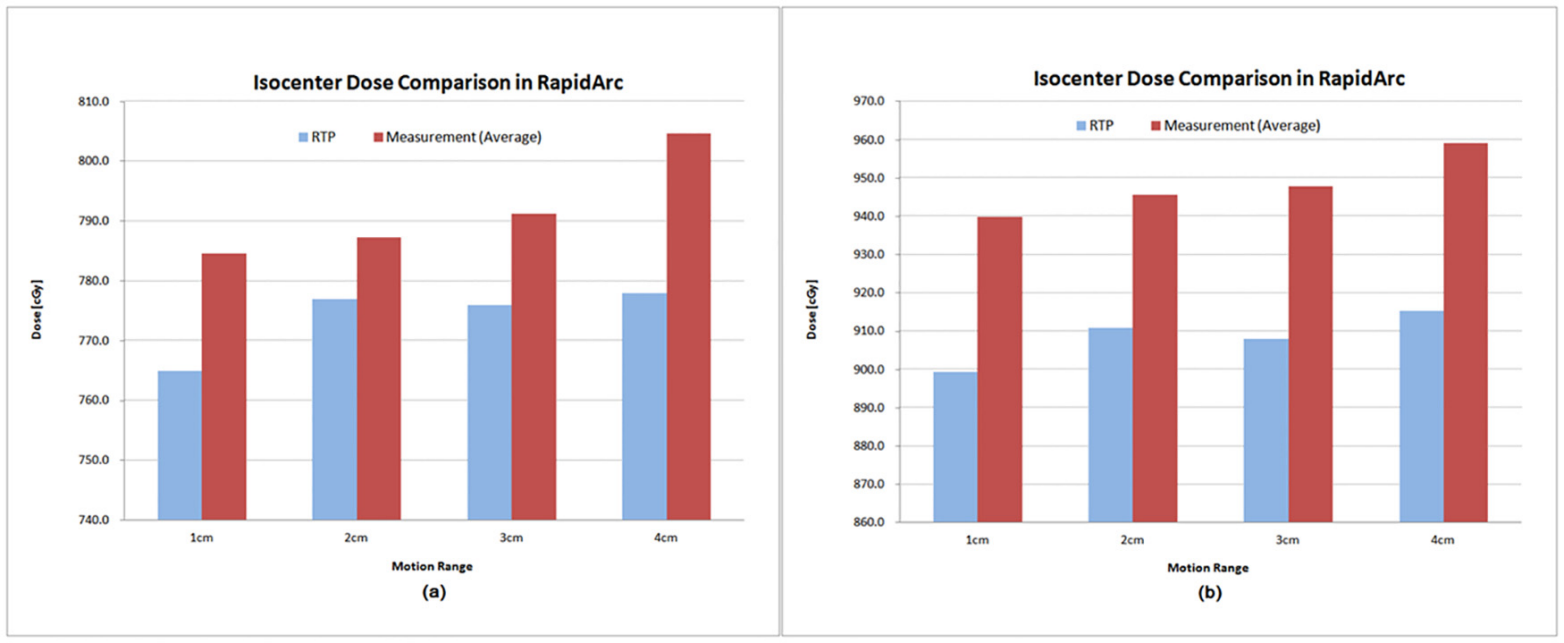
Comparison of RapidArc isocenter dose between RTP calculation and measurement with ion chamber under moving conditions. (a) tumor target in the peripheral region of left lung, (b) tumor target in the central region.

**Fig 11:**
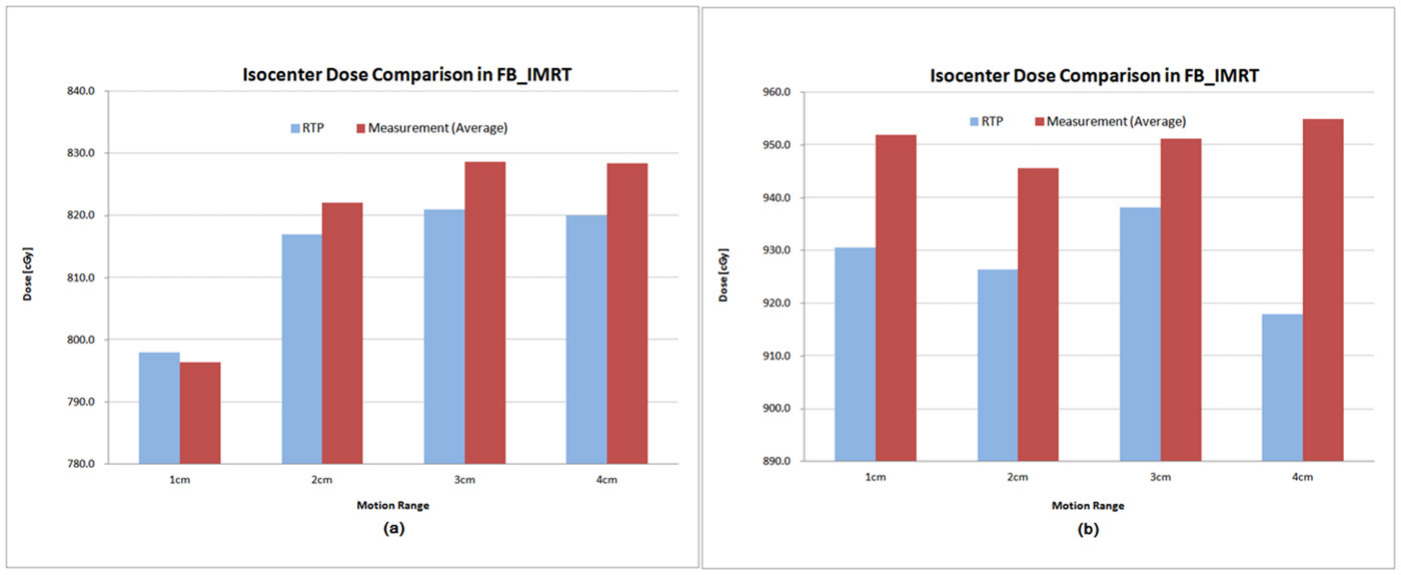
Comparison of FB_IMRT isocenter dose between RTP calculation and measurement with ion chamber under moving conditions. (a) tumor target in the peripheral region of left lung, (b) tumor target in the central region.

The measured isocenter dose was 3.3% higher on average in the RapidArc DQA and 1.5% higher on average in the FB_IMRT DQA than the calculated dose in the planning system. The difference between measured dose and calculated dose increased with an increase in the motion range and no dependence was found on the motion cycle. Figs 10 and 11 show that the average dose in the central region of PTV measured under the moving conditions was higher than the calculated dose in both the IMRS methods. The increase was greater in the RapidArc than in FB_IMRT and a similar trend was seen in the analysis of recalculated doses using 3DVH program.

## Discussion

The respiratory gating method can be used to reduce the OAR dose by reducing the treatment field size and increases the reliability based on a smaller difference between the calculated dose distribution and real treatment dose. Theoretically, the IMRT using respiratory gating has an advantages in delivering the prescribed dose distribution over the IMRT based on the ITV. However, for this to be realized clinically, the following conditions are required: accurate MLC operation, precise gating system, stable respiration of patient, and consistent organ motion correlation with respiratory signal. When a longer treatment time in the application of the gating system is considered, gating IMRT is not a superior method for correcting the respiratory motion effect clinically [17–19]. In addition, this method cannot be applied easily for lung cancer patients with deteriorated lung functions, and the more stable patient respiration pattern is required in the RapidArc treatment, which delivers the treatment with a continuous rotation of the heavy gantry and delicate motion of the MLC simultaneously. For patients who cannot maintain a stable respiratory pattern, the ITV based IMRT plan is more suitable, as the target volume includes the whole breathing motion range. One of its limitations is that, the IMRT plan does not calculate the real volume of the tumor target and OAR, and it is difficult to evaluate the real delivered dose under the moving condition.

In order to solve this problem, a practical method that can calculate the dose in the real volume of tumor target and OAR under the respiratory moving condition was devised in this study. The DQA data for the lung IMRS plans under the simulated respiratory moving condition was applied to 3DVH program and the dose in the real volume of tumor target and OARs was recalculated in accordance with motion ranges and cycles.

The tumor target dose thus calculated was higher than the calculated dose in the plan and a higher difference was shown in a RapidArc treatment compared to FB_IMRT. The increased dose to the real volume of PTV can be due to the continuous irradiation in the large IMRS treatment area including the whole motion range. An increased dose in RapidArc may arise from the continuous IMRS irradiation during the whole motion of tumor target. Although the aspect can be dissimilar in the different RapidArc plans, the irradiation condition in RapidArc can generally deliver a higher dose to the tumor target than the FB_IMRT, which irradiates with the fixed gantry angle beams. The results of this study show that the irradiated IMRS dose in the real tumor target volume under the moving condition is sufficiently higher than the prescribed dose and the RapidArc treatment delivers a higher and homogeneous dose distribution within the tumor target compared to FB_IMRT method.

The higher dose was also recalculated in the almost OAR compared with the dose in the original plan and the higher dose in a RapidArc treatment than FB_IMRT was also shown similarly with the tumor target case. The reason of higher recalculated dose in OAR seems to be similar with the tumor target case. The difference is that MLC motion is designed to minimize dose in the case of OAR in contrast to the intensive dose irradiation in tumor target case, that makes variable dose difference of OAR according to the location and volume size of OAR and the weight of dose constraints in the optimization process. Especially, the dose of OAR close to the moving tumor target could increase severely, which can be verified in the analysis results of centrally located tumor target in this study. Therefore, it is required to intensify the dose constraints of the OAR for the optimization process in the ITV based IMRS plan.

In conclusion, the tumor target dose in the ITV based IMRS treatment can get the sufficient dose more than a prescription and the respiratory motion is not critical as far as dose coverage of the tumor is concerned. However, in the ITV based IMRS treatment, an OAR can be irradiated with higher dose than the constraint dose specified in the optimization process. Hence for OARs that experience severe side effects with very high doses, such as the spinal cord, lower dose should be used as a severe constraint condition.

The accuracy of the dose calculation algorithm in the 3DVH program was not analyzed separately in this study because many studies have already verified its accuracy and feasibility [20–22]. Additionally, absolute point dose in the isocenter under the motion conditions was also measured. The higher value of the measured dose under the moving condition and higher degree of increase in RapidArc case are similar to the results from 3DVH calculations. These results prove the reliability of the dose calculations using the 3DVH program and the effectiveness of the devised method in this study. The gel dosimetry method [23] will be investigated for the future study to verify the dosimetric accuracy of the devised method in this study which can compare the recalculated dose in the 3DVH program with the measured gel dose in three dimensions.

## Conclusions

In this study, a practical method to calculate the dose distribution in the real volume of tumor target and OAR was devised for the analysis of the respiratory organ motion effect in the lung IMRS planned with the ITV including the whole tumor motion range. In the analysis with the humanoid chest phantom, a dose higher than the calculated dose in the plan was shown in both the tumor target and OAR under the moving conditions. However, the quantitative increase varied depending on the IMRS method used (RapidArc or FB_IMRT), location of OAR from the moving tumor target, and the constraint condition in the optimization process.

The method devised in this study for dose recalculation in the real volume of an organ under the respiratory motion condition can be applied effectively to analyze the real dose distribution in various IMRT cases planned using the ITV.
